# Traumatic Brain Injury Leads to Development of Parkinson's Disease Related Pathology in Mice

**DOI:** 10.3389/fnins.2016.00458

**Published:** 2016-10-13

**Authors:** Daniela Impellizzeri, Michela Campolo, Giuseppe Bruschetta, Rosalia Crupi, Marika Cordaro, Irene Paterniti, Salvatore Cuzzocrea, Emanuela Esposito

**Affiliations:** ^1^Department of Chemical, Biological, Pharmaceutical, and Environmental Sciences, University of MessinaMessina, Italy; ^2^Department of Pharmacology and Physiology, Saint Louis UniversitySt. Louis, MO, USA

**Keywords:** chronic traumatic brain injury, Parkinson's disease, α-synuclein, nuclear factor-κB, microglia, astrocytes, neurotrophic factors, non-motor symptoms

## Abstract

Traumatic brain injury (TBI) is a major health and socio-economic problem that affects all societies. This condition results from the application of external physical strength to the brain that leads to transitory or permanent structural and functional impairments. Moreover, TBI is a risk factor for neurodegeneration and can e.g., increase the risk for Parkinson's disease (PD), a late-onset neurodegenerative disorder with loss of dopaminergic neurons in substantia nigra. In this study, we wanted to explore the possible development of PD-related pathology within the context of an experimental model of TBI. Traumatic brain injury was induced in mice by controlled cortical impact. At different time points behavioral tests (open field, elevated plus maze tests, and Barnes maze) were performed: The animals were sacrificed 30 days after the impact and the brains were processed for Western blot and immunohistochemical analyses. Following TBI there was a significant decrease in expression of tyrosine hydroxylase and dopamine transporter in the substantia nigra as well as significant behavioral alterations. In addition, a strong increase in neuroinflammation was evident, as shown by increased levels of cyclooxygenase-2 and inducible nitric oxide synthase as well as IκB-α degradation and nuclear-κB translocation. Moreover, neurotrophic factors such as brain-derived neurotrophic factor, neurotrophin-3, nerve growth factor, and glial cell line-derived neurotrophic factor were decreased 30 days post-TBI. Interestingly, we observed a significant accumulation of α-synuclein in microglia compared to astrocytes. This study suggests that PD-related molecular events can be triggered upon TBI. The biological mechanisms linking brain trauma and neurodegenerative diseases need to be further investigated.

## Introduction

Traumatic brain injury (TBI) is described as an intracranial injury to the brain resulting from an external force that can lead to transient or permanent impairment of cognitive, physical, and psychosocial functions (Maas et al., 2008). Although TBI is basically considered an acute injury, the accumulation of clinical and laboratory verification has recognized the chronic pathology of the disease (Lozano et al., 2015). TBI is not a distinct event but a complex process (Masel and DeWitt, 2010) which causes structural and functional deficits due to both primary and secondary injury mechanisms (Davis, 2000). The primary injury results from the immediate mechanical disruption of cerebral tissue (acute stage) that occurs at the time of external force and includes contusion and hemorrhage (Gaetz, 2004). Secondary injury progresses over minutes to months after the primary damage and is the consequence of cascades of molecular and cellular events that lead to tissue damage, atrophy and brain cell death (Marklund et al., 2006; Bramlett and Dietrich, 2007; Lozano et al., 2015). TBI is closely related to the development of chronic neuro-inflammation, sensory-motor, and cognitive complications which appear some 5–6 years after the traumatic event, and corresponds to ~30 days in the mouse (chronic stage) (Ettenhofer and Abeles, 2009; Ozen and Fernandes, 2012; Acosta et al., 2013; Kirkwood and Yeates, 2013).

Few studies have investigated the possibility that chronic TBI causes secondary injury with pathophysiological changes similar to those seen in neurodegenerative diseases such as dementia pugilistica, Alzheimer disease (Saing et al., 2012) and Parkinson's disease (PD) (Saing et al., 2012; Acosta et al., 2013; Xiong et al., 2013). Parkinson's disease is a late-onset age-related neurodegenerative disorder of unknown etiology, characterized by a progressive loss of cathecolaminergic neurons, especially dopaminergic neurons within the substantia nigra pars compacta and the presence of α-syn-rich cytoplasmic neuronal inclusions named Lewy bodies (Dauer and Przedborski, 2003; Brandt et al., 2008). Alpha-synuclein (α-syn) is a 14-kDa synaptic protein with a key role in PD progression (Maroteaux et al., 1988) and it has a high propensity to misfold and aggregate as a response to an increase in its concentration, post-translational modifications, mutations, oxidative stress, pH, and metal ions (Hoyer et al., 2002; Jomova et al., 2010; Oueslati et al., 2010).

Neuroinflammation plays a fundamental role in the pathogenesis of TBI, involving cells of the innate immune system such as microglia, astrocytes, pro-inflammatory cytokines, and chemokines that interfere with the brain's endogenous ability to self-repair, thereby exacerbating neuronal cell death (Tajiri et al., 2014). The transcription factor nuclear factor kappa B (NF-κB) is a key modulator in inflammatory gene expression in the nervous system (Grilli and Memo, 1999), which is activated in response to inflammatory mediators and oxidative stress (Baldwin, 1996). Brains from patients with dementia pugilistica exhibit an abnormal accumulation of α-synuclein (α-syn) in axonal swellings and dystrophic neurites, suggesting a potential link between brain trauma, and the development of α-syn pathology in PD (Newell et al., 1999). The mediators between brain trauma and PD pathophysiology are unknown, but may involve oxidative and/or nitrative stress (Mésenge et al., 1998).

The present study was carried out to better understand the molecular mechanisms that associate the long-term consequences of TBI with the development of parkinsonism, focusing on the role of neuroinflammation and its impact on behavior in mice.

## Materials and methods

### Animals

Male CD1 mice (25–30 g, Harlan, Milan, Italy), aged between 10 and 12 weeks, were used for all studies. Mice were housed five per cage and maintained under a 12:12 h light/dark cycle at 21 ± 1°C and 50 ± 5% humidity. Standard laboratory diet and tap water were available *ad libitum*. The University of Messina Review Board for the care of animals approved the study. Animal care was in compliance with Italian regulations on protection of animals used for experimental and other scientific purposes (Ministerial Decree 16192) as well as with the Council Regulation (EEC) (Official Journal of the European Union L 358/1 12/18/1986).

### Controlled cortical impact (CCI) experimental TBI

TBI was induced in mice by a controlled cortical impactor (CCI) as previously described (Campolo et al., 2014). A craniotomy was induced in the right hemisphere, with a Micro motor hand piece and drill (UGO Basile SRL, Comerio Varese, Italy), encompassing bregma and lambda, and among the sagittal suture and the coronal ridge. The consequential bone flap was removed and the craniotomy enlarged additionally with cranial rongeurs (New Adalat Garh, Roras Road, Pakistan). A cortical contusion was made on the exposed cortex using the controlled impactor device Impact OneTM Stereotaxic impactor for CCI (Leica, Milan, Italy). Concisely, the impacting shaft was extended, and the impact tip was lowered over the craniotomy site until it touched the dura mater. Subsequently, the rod was retracted, and the impact tip was advanced farther to produce a brain injury of moderate severity for mice (tip diameter: 4 mm; cortical contusion depth: 3 mm; impact velocity: 1.5 m/sec). Instantaneously after injury, the skin incision was secure with nylon sutures, and 2% lidocaine jelly was spread to the lesion site to reduce pain.

### Experimental groups

Mice were randomly allocated into the following groups:

*Sham group*: mice were subjected to equal surgical procedures except for TBI and were kept under anesthesia for the duration of the experiment (*n* = 10).
*TBI group*: mice were subjected to CCI (*n* = 10).

Mice were sacrificed 30 days after TBI, the brains removed and the midbrain dissected out for study. Immunohistochemical localization of PD markers (TH, α-syn, and dopamine transporter (DAT) was assessed. Western blot was used to determine the translocation of NF-κB and degradation of IκBα. In addition, over the 30-day experimental period behavioral changes at 1, 7, 14, and 30 days were evaluated.

### Behavioral testing

All animals were subjected to the same battery of behavioral tests at 1, 7, 14, and 30 days post-CCI. All behavioral testing was conducted during the light cycle phase and in enclosed behavior rooms (50–55 dB ambient noise) within the housing room. The mice were placed in behavior rooms 5 min for 2 days for acclimation prior to the onset of behavioral testing.

The behavioral tests were conducted by three different reliable expert observers blinded to the injury status of the animals. Tests are described below:

*Open field test (OF)*: Locomotor activity and anxiety-like behavior were monitored for 5 min using the open field test, a white Plexiglas box 50 × 50 cm with its floor divided into 16 squares. Four squares were defined as the center and 12 squares along the walls as the periphery. Each mouse was gently placed in the center of the box and activity was scored as a line crossing when a mouse removed all four paws from one square and entered another. Before each trial, the chamber was cleaned with water containing a detergent. The animals' behavior was videotaped. The line crossings and the time spent in the center were counted and scored (Prut and Belzung, 2003).
*Elevated pluz-maze (EPM)*: The EPM test was used to measure anxiety-like behavior, as described previously (Pellow et al., 1985). The EPM apparatus consisted in two open arms (30 × 5 × 0.25 cm) and two enclosed arms (30 × 5 × 15 cm) extended from a common central platform (5 × 5 cm) and the entire apparatus was elevated by a single central support to a height of 60 cm above floor level. The behavioral model was based on rodents' aversion of open spaces that leads to thigmotaxis. Anxiety reduction was indicated by an increase in the proportion of time spent in the open arms and an increase in the proportion of entries into the open arms. The total numbers of arm entries and number of closed-arm entries were used as measures of general activity.
*Barnes maze*: The Barnes maze is a less stressful alternative than the Morris water maze, and is a validated test often used to assess spatial learning and memory in rodents. The test consists of a circular surface with up to 20 circular holes around its circumference. Under one of the holes is an escape box which can be reached by the rodent through the corresponding hole on the table top. The model was based on rodents' aversion of open spaces, which motivates the test subject to seek shelter in the escape box. The performance was measured by number of errors the rodent makes and the rate of decline in the number of errors per trial was calculated to represent a learning curve (Barnes, 1979).

### Western blot analyses

Cytosolic and nuclear extracts were prepared as previously described (Esposito et al., 2012), with slight modifications. The ipsilateral hemisphere from each mouse after injury was collected and suspended in Buffer A containing protease inhibitors, homogenized for 2 min, then centrifuged at 1000 × g for 10 min at 4°C. Supernatants contained the cytosolic fraction. The pellets, containing enriched nuclei, were resuspended in Buffer B containing 1% Triton X-100, 150 mM NaCl, 10 mM Tris-HCl pH 7.4, 1 mM, ethylene glycol tetra-acetic acid, 1 mM, ethylenediaminetetraacetic acid, 0.2 mM phenylmethanesulfonylfluoride and protease inhibitors. After centrifugation for 30 min at 15,000 × g at 4°C, the supernatants containing nuclear proteins were stored at −80°C for further analysis. The expression of inducible nitric oxide synthase (iNOS), cyclooxygenase (COX)-2 and IκBα were quantified in cytosolic fractions. NF-κBp65 was quantified in nuclear fractions collected 30 days after TBI. The filters were probed with specific antibodies for iNOS (1:1000; BD Biosciences, Milan, Italy), COX-2 (1:1000; Cayman Chemicals, Tallinn Estonia), glial fibrillary acidic protein (GFAP) (1:2000; Santa Cruz Biotechnology, Heidelberg, Germany), NFκBp65 (1:500; Santa Cruz Biotechnology) and IκBα (1:500; Santa Cruz Biotechnology) at 4°C overnight in 1 × phosphate-buffered saline (PBS), 5% (w/v), non-fat dried milk and 0.1% Tween-20. Membranes were incubated with peroxidase-conjugated bovine anti-mouse IgG secondary antibody or peroxidase-conjugated goat anti-rabbit IgG (1:2000; Jackson ImmunoResearch, West Grove, PA, USA) for 1 h at room temperature. To ascertain that blots were loaded with equal amounts of protein, then they were incubated in the presence of antibodies against β-actin or lamin A/C (1:5000; Santa Cruz Biotechnology). The signals were detected with enhanced a chemiluminescence detection system reagent according to the manufacturer's instructions (Super Signal West Pico Chemiluminescent Substrate, Pierce Thermo Scientific, Rockford, IL. USA). Relative expression of bands for IκBα (approximately 37 kDa), NF-κB (approximately 65 kDa), iNOS (approximately 130 kDa), COX-2 (approximately 72 kDa) and GFAP (approximately 55 kDa) were imported to analysis software (Image Quant TL, v2003) and standardized to β-actin and lamin A/C levels. The relative expression of the protein bands was calculated by densitometry with Bio-Rad ChemiDoc™ XRS+software. Molecular weight standards (10–250 kDa) were used to define molecular weight positions, and as reference concentrations for each protein.

### Immunofluorescence

After deparaffinization and rehydration, detection of TH, α-syn, NeuN, GFAP, Iba1, and neurotrophin-3 (NT-3) was carried out after boiling the tissue sections in 0.1 M citrate buffer for 1 min. Non-specific adsorption was minimized by incubating in 2% (vol/vol) normal goat serum in PBS for 20 min. Sections were incubated with one of the following primary antibodies: mouse monoclonal anti-TH (1:100, Millipore), mouse monoclonal anti-α-syn (1:100, Santa Cruz Biotechnology), rabbit polyclonal anti-GFAP (1:100, Santa Cruz Biotechnology), rabbit polyclonal anti-Iba1 (1:100, Santa Cruz Biotechnology), rabbit polyclonal anti-NeuN (1:100, Santa Cruz Biotechnology) or rabbit polyclonal anti-NT3 (1:100, Millipore) in a humidified oxygen and nitrogen chamber overnight at 37°C. Sections were then incubated with secondary antibody: fluorescein isothiocyanate-conjugated anti-mouse Alexa Fluor-488 (1:2000, Molecular Probes, Monza, Italy) or Texas Red-conjugated anti-rabbit Alexa Fluor-594 (1:1000, Molecular Probes) for 1 h at 37°C. For nuclear staining, 2 μg/ml 4′, 6′-diamidino-2-phenylindole (DAPI; Hoechst, Frankfurt, Germany) in PBS was added. Sections were observed at 20 × and 40x magnification using a Leica DM2000 microscope (Leica, Milan, Italy). TH^+^, a-syn^+^, NeuN^+^, GFAP^+^, Iba1^+^, and NT-3^+^cells were counted stereologically on sections cut at a 40 μm thickness and every 4th section was counted using a grid of 100 × 100 μm (Mallajosyula et al., 2008). Optical sections of fluorescence specimens were obtained using a HeNe laser (543 nm), an ultraviolet laser (361–365 nm) and an argon laser (458 nm) at a one-mi, 2 s scanning speed with up to eight averages; 1.5 μm sections were obtained using a pinhole of 250. Examining the most brightly labeled pixels and applying settings that allowed clear visualization of structural details, while keeping the highest pixel intensities close to 200, established contrast and brightness. The same settings were used for all images obtained from the other samples that had been processed in parallel. Digital images were cropped and figure montages prepared using Adobe Photoshop 7.0 (Adobe Systems; Palo Alto, California, United States). The co-localization of images was examined with Image J software (National Institutes of Health) as described previously (Zhou et al., 2011).

### Immunohistochemistry

Brain tissues were fixed in 10% (w/v) buffered formaldehyde 30 days after TBI, and 7-μm sections were prepared from paraffin-embedded tissues. After deparaffinization, endogenous peroxidase was quenched with 0.3% H_2_O_2_ in 60% methanol for 30 min. The sections were permeabilized with 0.1% Triton X-100 (Sigma-Aldrich, Milan, Italy) in PBS (pH 7.4) for 20 min. Non-specific adsorption was minimized by incubating the section in 2% normal goat serum in PBS for 20 min. Endogenous biotin- or avidin-binding sites were blocked by sequential incubation for 15 min with avidin and biotin. The sections were then incubated overnight with one of the following primary antibodies diluted in PBS: anti-TH (1:500, Millipore-monoclonal or polyclonal), anti-DAT (1:500, Santa Cruz Biotechnology polyclonal), polyclonal anti-glial cell line-derived neurotrophic factor (GDNF) (1:500, Santa Cruz Biotechnology), polyclonal anti-brain-derived neurotrophic factor (BDNF) (1:500, Santa Cruz Biotechnology), anti-α-syn (1:250, Santa Cruz Biotechnology-polyclonal), and anti-NT-3 (1:500, Millipore-polyclonal). The immunohistochemical pictures were collected by Zeiss microscope using Axio Vision software. For graphic representation of densitometric analyses, we measured the intensity of positive staining (brown staining) by computer-assisted color image analysis (Leica QWin V3, UK). The percentage area of immunoreactivity (determined by the number of positive pixels) was expressed as percent of total tissue area (red staining). Photomicrographs were assessed densitometrically with Optilab software (Graftek, Mirmande, France) on a MacBook Pro computer (Apple, Cupertino, CA, USA). Analysis was carried out by assigning quantitative different criteria for staining intensity as described by Ding et al. (2012), which included assignment of staining intensity using a scale of 0–10 (with 0 indicating a lack of brown immunoreactivity and 10 reflecting intense dark brown staining) by three different reliable expert observers. The mean was then calculated and results converted into grades: a score of 1–3 was assigned “+,” 4–6 was “++,” more than 7 was “+++”(Ding et al., 2012). Scores from all sections of each brain were averaged to give a final score for each mouse. All histological studies were performed in a blinded fashion.

### Tissue processing and histology

An experienced histopathologist evaluated sagittal sections of 5-μm thickness from the perilesional brain area of each animal. Histopathologic changes of the gray matter were scored on a six-point scale (Kawai and Akira, 2007) as described by Campolo et al. (2014). Scores from all sections of each brain were averaged to give a final score for each mouse. All histological studies were performed in a blinded fashion.

### Statistical analysis

All values in the figures and text are expressed as mean ± standard error of the mean (SEM) of N observations. For *in vivo* studies N represents the number of animals. In those experiments involving histology or immunohistochemistry, the figures shown are representative of at least three experiments performed on different days. Results were analyzed by one-way ANOVA followed by a Bonferroni *post-hoc* test for multiple comparisons. A F-value was shown, *p*-value of less than 0.05 was considered significant.

## Results

### Changes of PD markers in the SNc after chronic TBI

**Figure 1:**
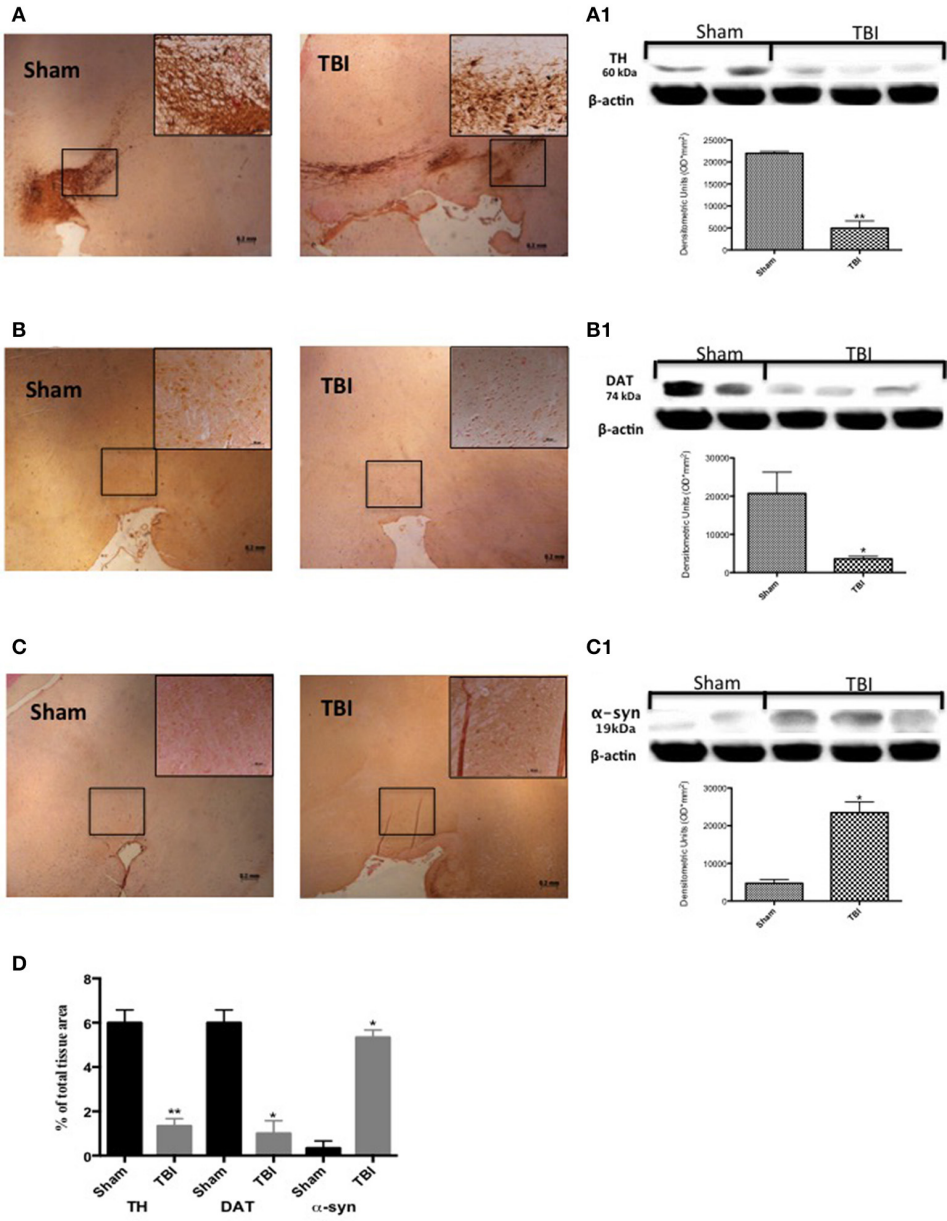
**Effect of chronic TBI on Parkinsonian markers**. Midbrain was stained with antibodies against tyrosine hydroxylase **(A)**, dopamine transporter **(B)** and α–synuclein **(C)**. Immunohistochemical analysis of midbrain obtained from mice subjected to TBI revealed a positive staining for TH, DAT, and α-syn [TBI panels TBI **(A–C)** respectively; see densitometric analysis, **D**] compared with sham-operated mice [Sham panels TBI **(A–C)** respectively; see densitometric analysis, **D**]. Data are expressed as a percentage of total tissue area and are means ± SE of 5 mice/group. ^**^P < 0.005 vs. Sham; ^*^P < 0.05 vs. sham (Student's *t*-test). Western blot analysis confirmed our data (**A1–C1** respectively). Each data are expressed as Mean ± SEM from *N* = 5 mice/group. ^*^P < 0.01 vs. sham, ^**^P < 0.005 vs. sham.

To examine if chronic TBI can modify PD-like markers, brain sections from control and TBI mice 30 days after surgery were stained with dopaminergic-specific markers (TH and DAT) and α-syn. Midbrain expression of TH-positive neurons and DAT was significantly decreased 30 days after TBI (Figures 1A,B, respectively, see densitometric analysis, Figure 1D; *F* = 3 for TH and for DAT). Western blot analysis confirmed a significant reduction of TH and DAT protein expression (Figure 1A1, *P* < 0.005; and Figure 1B1, respectively, *P* < 0.05; *F* = 1.111 for TH and 4.88 for DAT). In contrast, chronic TBI resulted in a visible increase in α-syn staining compared to control group (Figure 1C, see densitometric analysis, Figure 1D, *F* = 3) and protein as shown by Western blot analysis (Figure 1C1 *P* < 0.01; *F* = 0.078).

**Figure 2:**
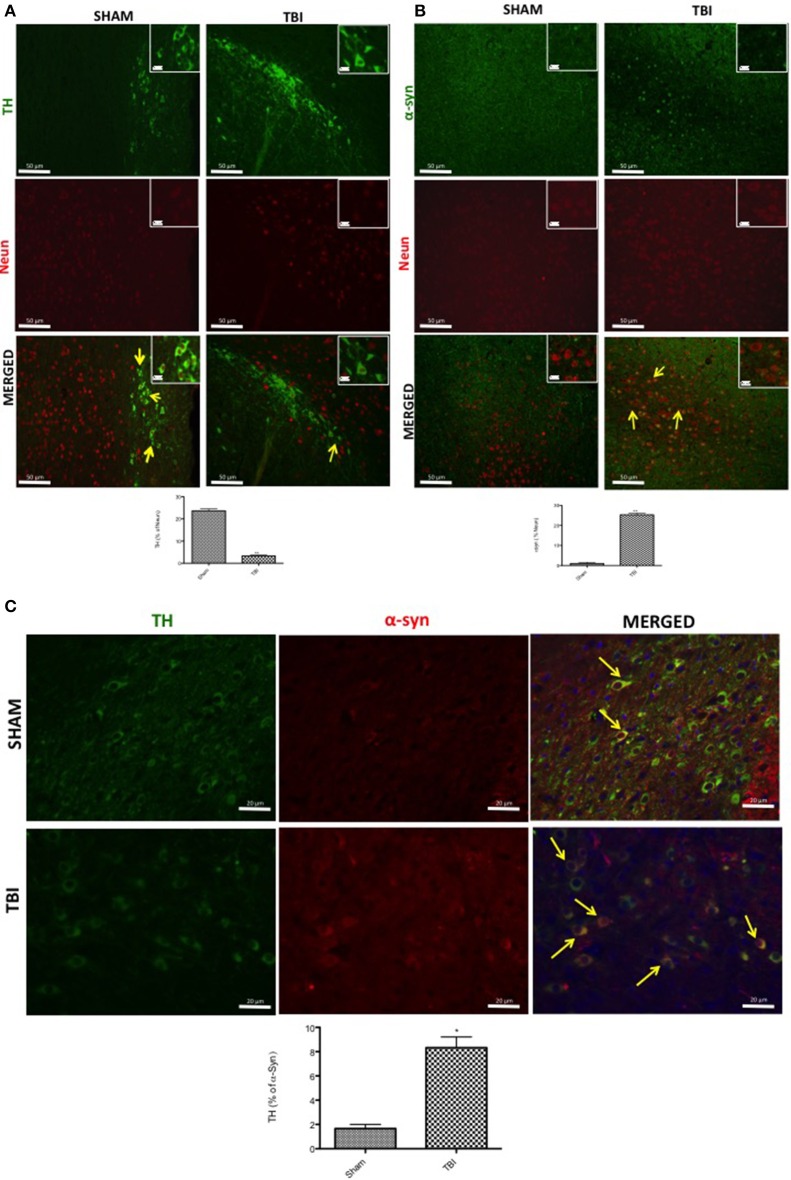
**Effects of chronic TBI on Tyrosine hydroxylase (TH) and α-synuclein expression in NeuN positive cells**. Midbrain was double-stained with antibodies against NeuN (red), TH (green) **(A)**, or α-syn (red) **(B)**. The red spots indicate the co-localizations (merged) indicated by yellow arrows. TH-positive neurons were significantly reduced after chronic TBI (TBI merged **A**). Midbrain sections revealed increased α-syn positive neurons 30 days after TBI (TBI merged **B**). Moreover, midbrain sections showed a marked α-syn accumulation in TH^+^ cell (TBI merged **C**). Scale bar = 50 and 20 μm (particle). Data are expressed as Mean ± SEM from *N* = 5 mice/group. Counting of colocalized cell confirmed our data. The co-localization of image was analyzed with image J software. ^*^P < 0.05 vs. sham (Student's *t*-test), ^**^P < 0.005.

To confirm these data, TH and α-syn were double-stained with antibodies against the neuronal marker NeuN, in the midbrain. Thirty days after TBI there was an evident accumulation of α-syn in neurons compared with sham mice (Figure 2B, see corresponding cell count analysis; *F* = 0.428). On the other hand, TH-positive neuron counts were significantly decreased (Figure 2A, see corresponding cell count analysis; *F* = 7). Also, to evaluate the α-syn accumulation specifically in the dopaminergic neurons we assessed a double staining between TH and α-syn (Figure 2C). We noticed a basal level of α-syn into the dopaminergic neurons TH-positive in sham group while 30 days after TBI there was an increasing accumulation of α-syn in the TH positive neurons (Figure 2C).

### Microglia, but not astrocytes in the SNc regulate α-syn expression after chronic TBI

**Figure 3:**
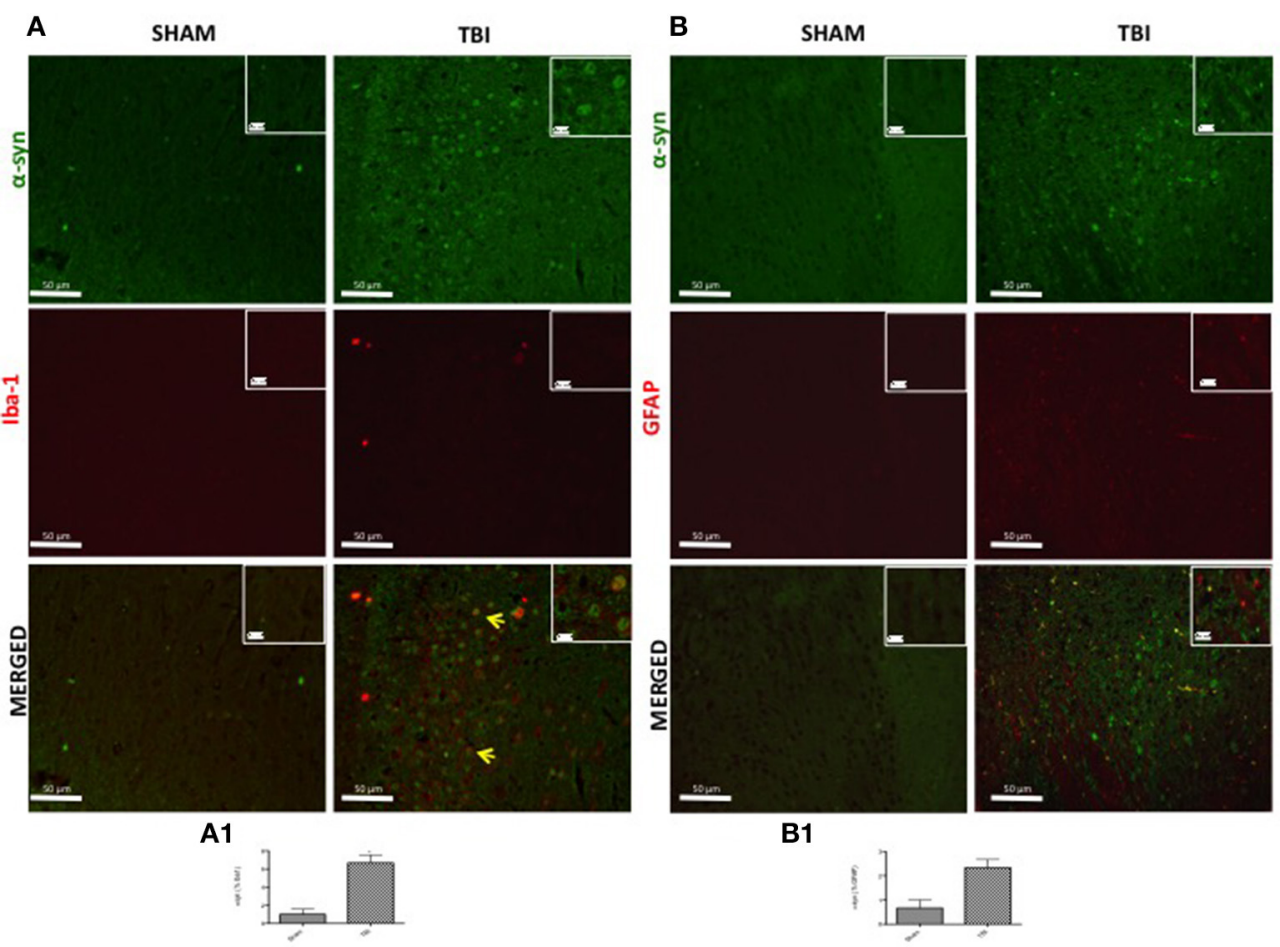
**Effect of chronic TBI on α-synuclein expression in ionized calcium binding adaptor molecule 1 (Iba1) and glial fibrillary acidic protein (GFAP) positive cells**. Midbrain sections wer double-stained with antibodies against Iba1 (red) or GFAP (red) and α-syn (green). The red spots indicate the co-localizations (merged) indicated by yellow arrows. There were increased microglia cells (Iba1^+^cells) in TBI mice **(A)** as compared to the control group (Sham **A**). α-syn positive neurons were significantly increased in microglia after chronic TBI (TBI merged **A**). Considerable astrogliosis (GFAP^+^cells) was present in TBI panels **(B)**. α-syn positive neurons did not show any significant immunoreactivity in astrocytes after TBI (TBI merged **B**). Scale bar = 50 and 20 μm (particle). Each data are expressed as Mean ± SEM from *N* = 5 mice/group. Counting of colocalized cell confirmed our data. The co-localization of image was analyzed with image J software. ^*^P < 0.05.

To evaluate microglia involvement and its correlation with PD markers, the midbrain was double-stained with antibodies against Iba1 (red; Figure 3A) and α-syn (green; Figure 3A). Microglial cells (Iba1-+cells) visibly expressed α-syn-positive staining in chronic TBI tissue (Figure 3A) compared to sham. Further, there was a clear co-localization of α-syn and Iba1 in the TBI samples (merged, Figure 3A- see cell count analysis A1; *F* = 0.428).

**Figure 4:**
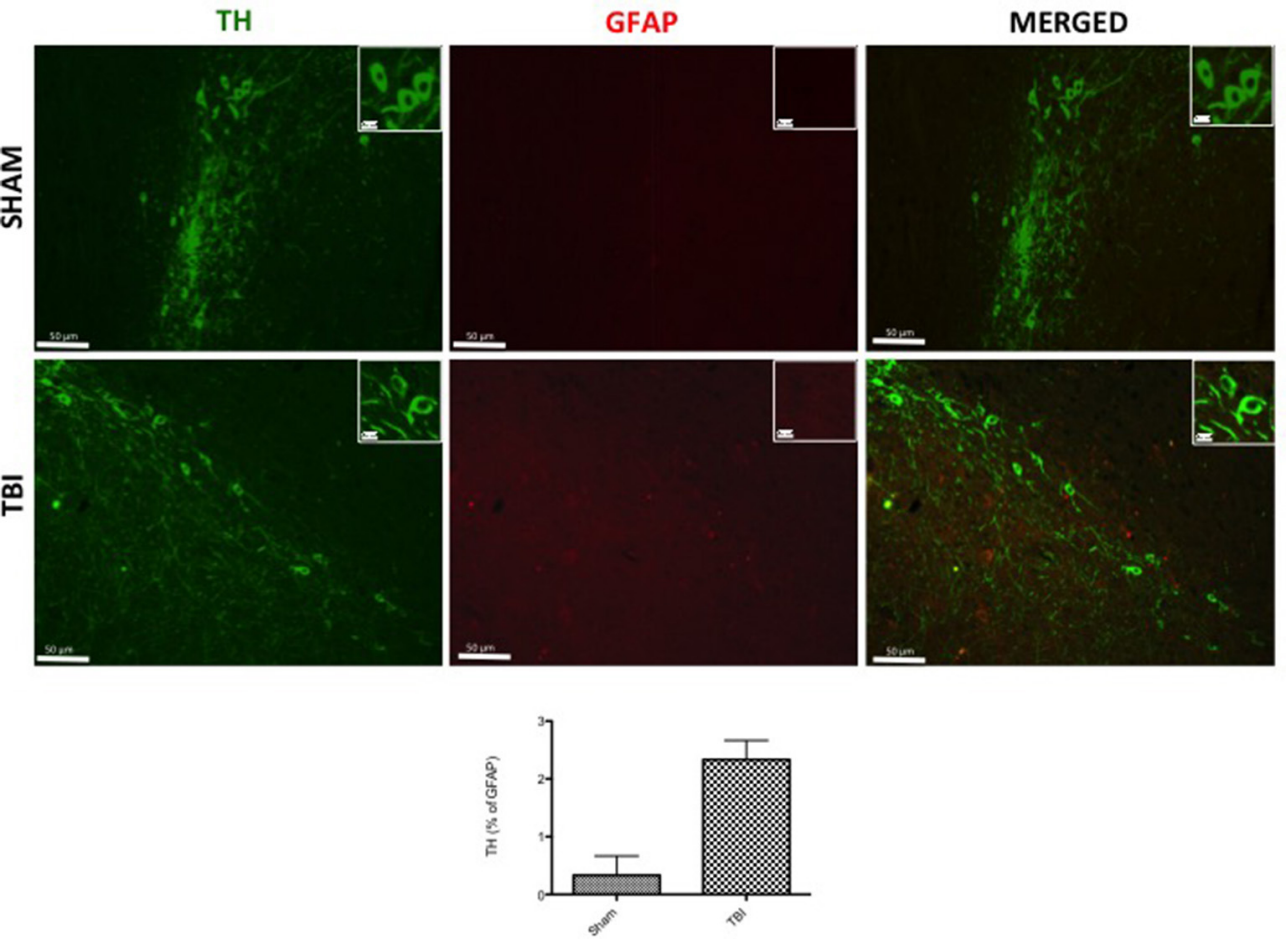
**Effect of chronic TBI on Tyrosine hydroxylase (TH) expression in glial fibrillary acidic protein (GFAP) positive cells**. Midbrain tissues were double-stained with antibodies against GFAP (red) and TH (green). The red spots indicate the co-localizations (merged) indicated by yellow arrows. Midbrain sections revealed increased astrogliosis (GFAP^+^ cells) in TBI panels. TH positive neurons were significantly reduced after TBI (TBI merged panel). Scale bar = 50 and 20 μm (particle). Each data are expressed as Mean ± SEM from *N* = 5 mice/group. Counting of colocalized cell confirmed our data. The co-localization of image was analyzed with image J software.

To analyze the activation of astrocytes and its involvement in PD, contused brain tissue at the collision site 30 days after injury was double-stained with for GFAP (red; Figure 3B) and α-syn (green; Figure 3B) or TH (green; Figure 4). Midbrain sections showed an increased astrogliosis (GFAP+ cells) in TBI panels (Figures 3B, 4). Furthermore, there was a non-significant co-localization of both α-syn and TH in GFAP^+^ cells after chronic TBI [merged, Figures 3B, 4, respectively- see cell count analysis in Figures 3B1 (*F* = 1) and Figure 4A, respectively (*F* = 1)].

### Chronic TBI induces a significant neuroinflammatory response regulated by NF-κB

**Figure 5:**
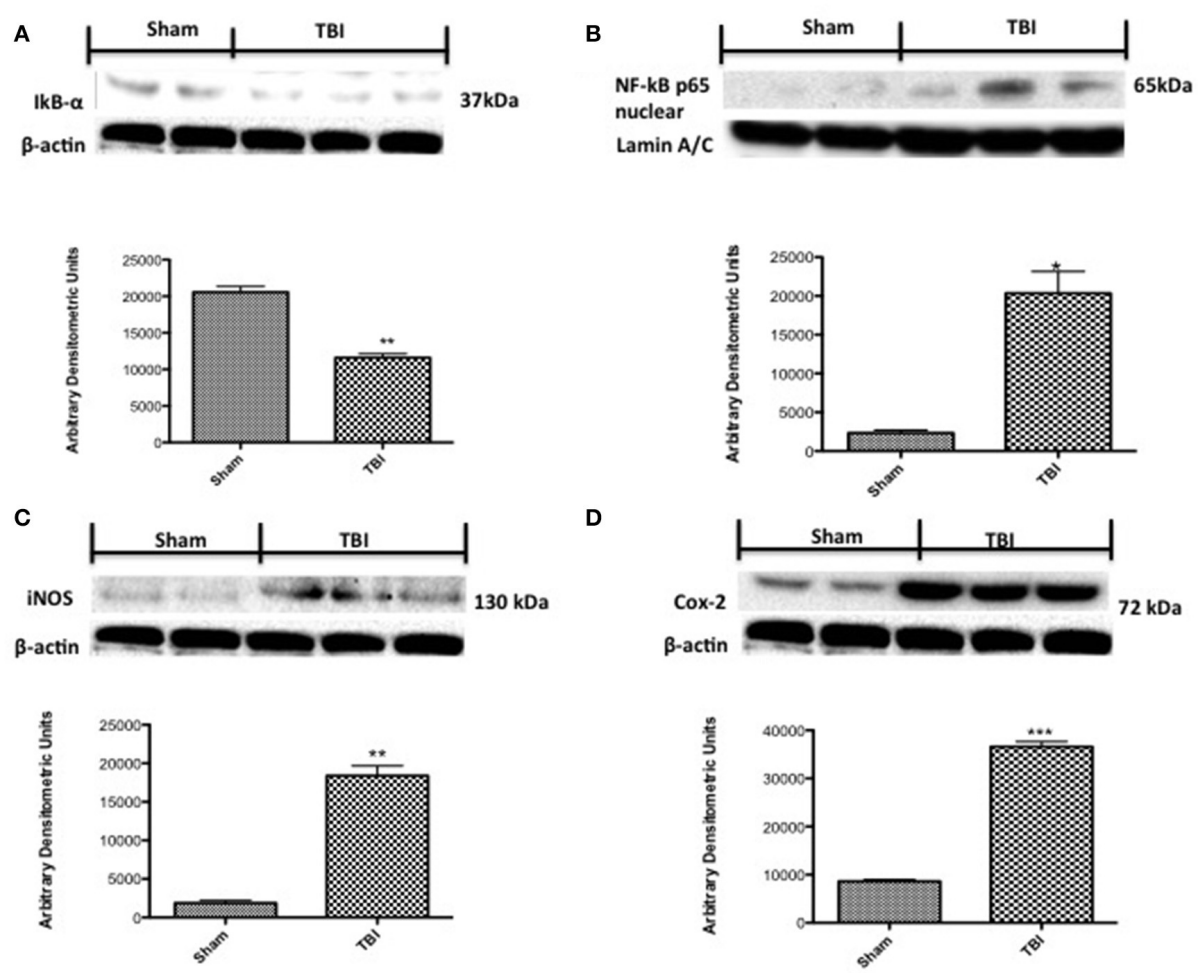
**Effects of chronic TBI on Nuclear factor κB (NF-κB) pathway and pro-inflammatory enzymes**. Degradation of IκBα was significantly increased 30 days after TBI **(A)**. Also, chronic TBI resulted in enhancednuclear translocation of p65 **(B)**. A significant increase in inducible nitric oxide synthase (iNOS) and cyclooxygenase (COX)-2 (**C,D**, respectively) was observed in the midbrain from TBI mice compared with the Sham mice (**C,D**, respectively). Data show one representative blot from three independent experiments with similar results. Data are expressed as Mean ± SEM from *N* = 5 mice/group. ^*^ *P* < 0.01 vs. sham, ^**^P < 0.005 vs. sham,^***^P < 0.005 vs. sham. (Student's *t*-test).

To investigate the cellular substrate(s) that link PD with chronic TBI, Western blot analysis was assessed in midbrain tissue 30 days post-TBI, using IκBα- and NF-κB- specific antibodies. There was a basal expression of IκBα in sham mice (Figure 5A, see densitometry analysis (*P* < 0.005), while IκBα expression was considerably reduced in mice subjected to chronic TBI (Figure 5A, see densitometry analysis, *P* < 0.005; *F* = 1.087). Moreover, p65 subunit translocation was increased in nuclear brain homogenates after TBI, compared with sham (Figure 5B, see densitometry analysis *P* < 0.01; *F* = 0.0195). Translocation of NF-κB is a critical phase in the coupling of extracellular stimuli to the transcriptional stimulation of specific pro-inflammatory target genes such as iNOS and COX-2. To evaluate the role of nitric oxide in TBI, iNOS expression was evaluated by Western blot analysis. At 30 days post-TBI there was a significant increase in iNOS expression in midbrain of TBI mice (Figure 5C, see densitometry analysis, *P* < 0.005, *F* = 0.0312). COX-2 expression was also stimulated by TBI compared to sham (Figure 5D, see densitometry analysis, *P* < 0.001, *F* = 0.0019).

### Chronic TBI reduces neurotrophic factor expression levels

**Figure 6:**
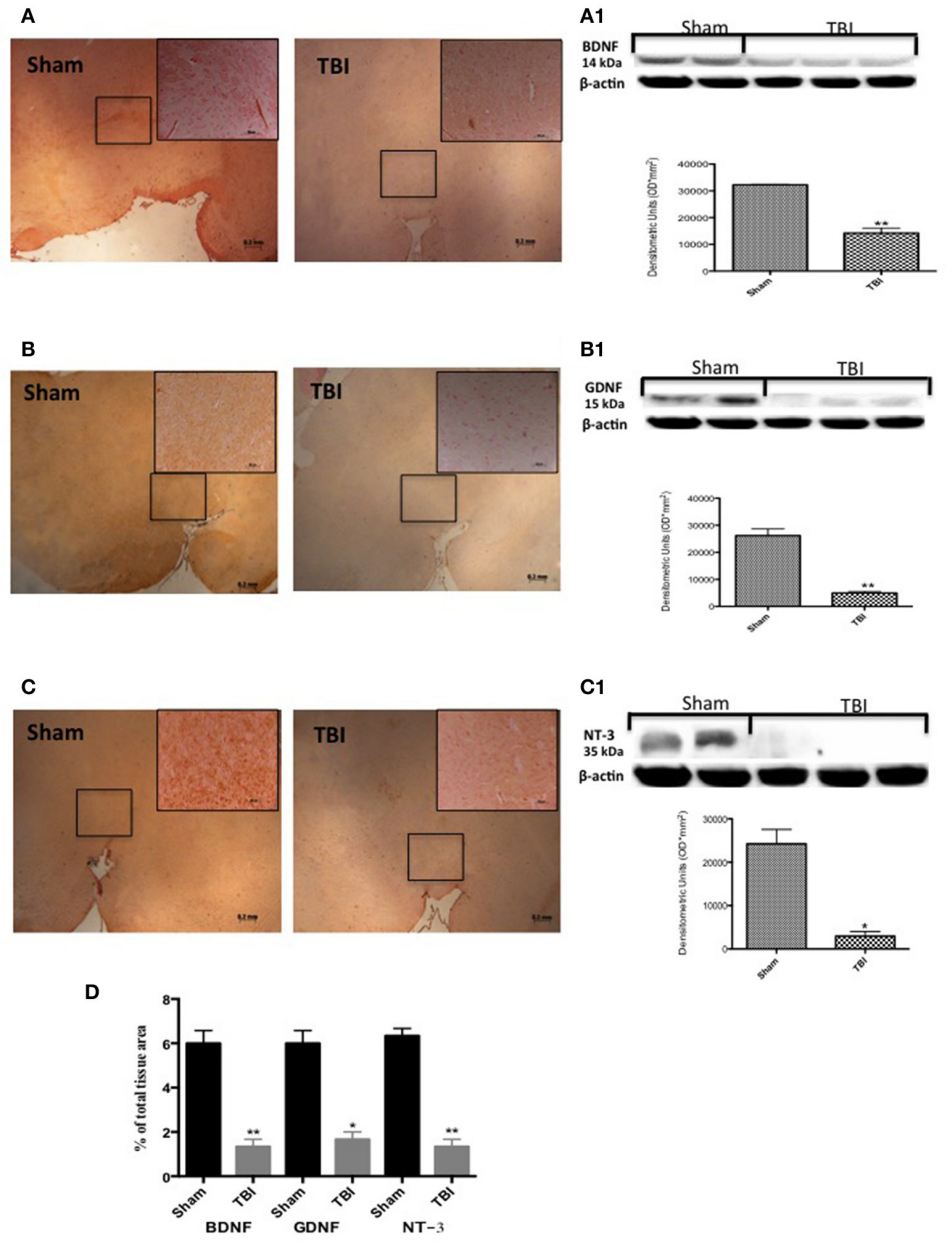
**Effect of chronic TBI on Neurotrophic factors**. By immunohistochemical analysis, a basal level of BDNF **(A)** GDNF **(B)** and NT-3 **(C)** positive staining was detected in midbrain samples from sham-operated mice (sham panels **A**–**C** respectively; see densitometric analysis, **D**). BDNF and GDNF and NT-3 expression was significantly reduced in midbrain samples from TBI mice (TBI panels **A**–**C** respectively; see densitometric analysis, **D**). Data are expressed as a percentage of total tissue area and are means ± SE of 5 mice/group. ^**^P < 0.005 vs. Sham; ^*^P < 0.05 vs. sham (Student's *t*-test). Western blot analysis confirmed our data showing a significant decrease of neurotrophic factor following TBI (**A1–C1** respectively, see correspond densitometric analysis). Each data are expressed as Mean ± SEM from *N* = 5 mice/group. ^**^P < 0.005 vs. sham; ^*^P < 0.05 vs. sham (Student's *t*-test).

To test whether chronic TBI modulates PD via regulation of neurotrophic factors, we used immunohistochemistry to examine BDNF, GDNF, and NT-3 levels in the midbrain. Thirty days post-trauma there was a reduction in immunostaining for all three neurotrophic proteins (Figures 6A–C, respectively, see densitometric analysis, Figure 6D; *F* = 3 for BDNF and GDNF, and *F* = 1.0002 for NT-3) in comparison to sham animals. Moreover, Western blot analysis confirmed these observations (Figures 6A1–C1, respectively, see densitometric analysis, Figure 6D: *P* < 0.005, *P* < 0.001, and *P* < 0.05, respectively; and *F* = 2.156, 1.318, 9.30 for BDNF, GDNF, and NT-3).

### Chronic TBI induces depression- and anxiety-like behaviors in the mouse

**Figure 7:**
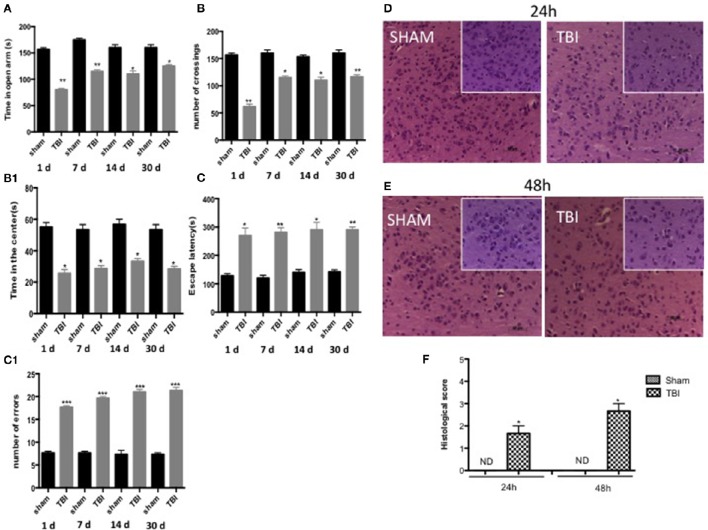
**Effect of chronic TBI on anxiety-like behaviors**. The degree of non-motor impairment was assessed in a blinded manner at different time points (1,7,14, and 30 d) by elevated plus maze (EPT), open field (OF), and Barnes maze. EPM tests showed that TBI animals spent less time exploring the open arms and head dipping than Sham animals **(A)**. No significant differences in rearing or grooming were observed. OF tests showed that TBI mice traveled significantly shorter distances and didn't stay in the center area for longer **(B,B1)**. Barnes maze revealed a decrease of spatial learning induced by TBI in all time point increasing the latency for escape **(C)** and the mean number errors **(C1)** Data are means ± SE of 10 mice/group. ^*^P < 0.05 vs. sham; ^**^P < 0.005; ^***^P < 0.001 vs. sham (ANOVA and Bonferroni test). H&E staining: **(D–E)**. As compared with sham groups at 24 and 48 h (**D,E** respectively, see histological score **F**), after TBI wasn't shown a significant alteration of tissue morphology (**D,E** respectively, see histological score **F**) in the midbrain. Each data are expressed as Mean ± SEM from *N* = 5 Mice for each group. Scale bar = 50 and 20 μm (higher magnification).

To examine anxiety-like behavior and locomotor function, mice subjected to chronic TBI were compared to sham mice at 1, 7, 14, and 30 days on several behavioral tests, including the open field test, EPM, and Barnes maze. Mice were tested in the EPM at all time points, TBI mice spent notably less time in the open arms as compared to sham animals (Figure 7A, see densitometric analysis, Figure 7F, *F* = 1.333 at 1 days, *F* = 1 at 3 days, *F* = 1 at 7 days, *F* = 4 at 30 days). The anxiety-like phenotype induced by the lesion was confirmed in the open field test (Figures 7B,B1, see densitometric analysis, Figure 7F; ^*^P < 0.05 and ^**^P < 0.005; Figure 7B, *F* = 1.750 at 1 days, *F* = 4 at 3 days, *F* = 3 at 7 days, *F* = 3 at 30 days; Figure 7B1, *F* = 1.531 at 1 d, *F* = 3.226 at 3 days, *F* = 4 at 7 days, *F* = 4 at 30 days). Chronic TBI mice showed a pronounced increase in thigmotaxis, seen as the tendency to remain close to the wall (Simon et al., 1994), when compared to sham mice, as specified by less time spent in the center of the open field (Figure 7B, ^*^P < 0.05 and ^**^P < 0.005 vs. sham) and shorter distance covered in the center of the open field (Figure 7B1, *P* < 0.05 vs. sham). Spatial learning and memory were assessed in TBI mice using the Barnes maze. In this test, sham mice quickly learned to escape the open field and reach the black escape box, as revealed by the rapid decline in escape latency. In contrast, there was a statistically significant decrease in spatial learning induced by TBI for all time points, with an increase in escape latency and mean number of errors (Figures 7C,C1, respectively, see densitometric analysis, Figure 7F, ^***^P < 0.001 vs. sham; Figure 7C) *F* = 12 at 1 days, *F* = 2.583 at 3 days, *F* = 7 at 7 days, *F* = 1.714 at 30 days): Figure C1) *F* = 1 at 1 days, *F* = 1 at 3 days, *F* = 2.333 at 7 days, *F* = 4 at 30 days).

Behavioral tests showed the degree of severity in our TBI model 30 days post-trauma. Using hematoxylin and eosin staining we failed to detect any frank cell loss or alteration of tissue morphology in the midbrain either 24 or 48 h after TBI compared to sham groups (Figures 7D,E, respectively, see densitometric analysis, Figure 7F, see histological score ^*^P < 0.05).

## Discussion

A strong association of TBI with an increased risk of PD has been documented (Bower et al., 2003; Goldman et al., 2006). The development of animal models of TBI that validate many fundamental pathophysiological processes related to PD has been an important advance in this field (Uryu et al., 2003; Xiong et al., 2013). Among the many mechanisms associated in PD pathology, α-syn accumulation coupled with TBI appears to synergistically impact on PD symptoms (Shahaduzzaman et al., 2013; Ulusoy and Di Monte, 2013). In the present study we demonstrate that already 30 days after TBI PD-like markers are significantly up regulated. In addition, midbrain tissue of chronic TBI mice displayed a remarkable decrease in the immunohistochemical expression of dopaminergic markers, along with an evident accumulation of α-syn in neurons.

Inflammation plays an important role in PD (Engler et al., 2009). Dopaminergic neurons are highly susceptible to inflammation and oxidative damage and neuroinflammation mediated by glial cells has attracted much attention. Previous studies revealed abnormalities in astrocytes and microglia in PD patients, suggesting involvement of these two cell populations in the development of early stage PD (Rappold and Tieu, 2010).

TBI is reported to lead to accumulation of α-syn in microglial cells and astrocytes^11^, leading to propagation of damage to neurons that perpetuates neurodegeneration. Our results show a significant accumulation of α-syn in microglia but not astrocytes, which could represent an important link between TBI and PD. In addition, microglial infiltration into the substantia nigra has been suggested to alter the inflammatory environment, and promote the buildup of α-syn (Acosta et al., 2015). In some studies microglia have been shown to activate NF-κB, leading to dopamine neuron degeneration, possibly through apoptotic cell death (Phani et al., 2012).

NF-κB is a cardinal transcriptional regulator of inflammation and apoptosis, neuronal cell survival, and signaling involved in brain damage. NF-κB activation may persist for at least 1 year following cortical injury, suggesting that chronic NF-κB activation may play a role in long-term inflammatory mechanism following brain trauma (Nonaka et al., 1999). In fact, our data show a clear and significant expression of NF-κB 30 days after TBI. In line with this, there was a significant increase in the degradation of cytoplasmic IKB. Further, NF-κB activation in the midbrain was accompanied by increased transcription of the inflammatory markers COX-2 and iNOS. The latter may result in production of high levels of nitric oxide and superoxide radicals, which are neurotoxic. COX-2, which generates prostaglandins, represents a potential hazard for dopamine neurons. In fact, increased COX-2 expression was localized to the substantia nigra pars compacta in post-mortem brains of PD patients (Przedborski, 2007). We hypothesize that NF-κB activity in the midbrain could play a key role in determining inflammatory processes in the lesioned nigrostriatal pathway, which consequently influence the neurodegenerative outcome.

Neurons in the substantia nigra show up-regulated dopaminergic markers in response to increases in BDNF, GDNF, and NT-3 (Peterson and Nutt, 2008). BDNF elevation has been associated to better performance into the motor cortex (Zhao et al., 2016). GDNF has been linked to the survival of dopaminergic cells in the substantia nigra through increases in synaptic excitability (Bourque and Trudeau, 2000) and the inhibition of apoptosis (Burke et al., 1998). BDNF, NT-3, and GDNF, by binding to their respective high-affinity receptors stimulate the survival and morphological differentiation of midbrain dopaminergic neurons and increase dopamine uptake (Baquet et al., 2005; von Bohlen und Halbach et al., 2005; Grandoso et al., 2007; Stahl et al., 2011) Interestingly, our study indicates a significant reduction in expression of BDNF, GDNF, and NT-3 in the midbrain 30 days after TBI.

Parkinson's disease does not only feature motor symptoms, but also non-motor symptoms which can lead to cognitive and psychiatric disturbances (Bonito-Oliva et al., 2014). The depressive-like phenotype observed in our TBI-PD-induced mouse model was paralleled by increased thigmotaxis and reduced time spent in the open arms of the EPM, two standard behavioral parameters of anxiety. These data are in agreement with the frequent co-morbidity between anxiety and depression observed in PD patients (Menza et al., 1993) and with a number of observations in experimental models. Also, human studies comparing neurobehavioral outcomes after TBI suggest that cognitive and emotional impairments increase with injury severity (Washington et al., 2012). Little is known about the mechanistic role, direct or indirect, of the substantia nigra on learning processes, and our Barnes maze test in chronic TBI mice could reveal early dysfunction within the substantia nigra (Li et al., 2013) or inability of the mice to effectively utilize a search strategy. Furthermore, our findings demonstrate that acute-to-chronic TBI could cause long-term deficits in cognitive function that accompanies gradual lesion expansion and continuing neurodegeneration evolving in PD.

The present observations provide the first demonstration that a cortical lesion can expand to the midbrain and culminate in the expression of PD-like elements 30 days post-trauma. Specifically, our study indicates specific cellular mechanisms that may link chronic TBI and PD, in particular neuroinflammatory reactions via the NF-κB pathway. Moreover, microglia appear to occupy a prominent position as a link between TBI and the development of PD-like pathology with aberrant α-syn accumulation, which could play an important role in the neurodegenerative process. In terms of the PD-related non-motor symptoms, chronic TBI enhanced anxiety-like behavior and spatial learning. Based on previous findings (DeKosky et al., 2013), our study also suggests that such PD-like pathology and symptoms could represent an etiology for other neurodegenerative diseases such as chronic traumatic encephalopathy (dementia pugilistica). Although there are limitations and challenges with animal models for mild TBI, these should still be relevant for the study of behavioral sequelae of single and repetitive mild TBI and how they relate to the numerous complex histopathological pathways. For example, the safety, and efficacy identified in animal studies may not generally translate to human trials, since one cannot exclude the possibility that some residual unmeasured confounders exist (e.g., a behavior that may lead a person to be more likely to sustain a TBI and/or that may also be an independent risk factor for PD). Also, this study is unable to provide information about the neuro-functional assessment of chronic TBI.

Regardless of its limitations, this study has investigated an under-appreciated biological mechanism that indicates a causal relationship between brain injury and neurodegenerative disease; In particular, our findings suggest that sustained neuroinflammatory processes following a single moderate TBI can lead to a PD-like pathophysiology in the midbrain.

## Author contributions

Study concept and design: DI, SC, and EE. Acquisition of data and statistical analysis: GB, RC, MaC, and IP. Analysis and interpretation of the data: DI, MiC, SC, EE. Drafting of paper: MiC and EE. Critical revision of the manuscript for intellectual content: SC, EE. All authors read and approved the final manuscript.

### Conflict of interest statement

The authors declare that the research was conducted in the absence of any commercial or financial relationships that could be construed as a potential conflict of interest. The reviewer SAH and handling Editor declared their shared affiliation, and the handling Editor states that the process nevertheless met the standards of a fair and objective review.

## Abbreviations

DAPI: 4′, 6′-diamidino-2-phenylindole
BDNF: Brain-derived neurotrophic factor
CCI: Controlled cortical impact
Cox-2: Cyclooxygenase-2
DAT: Dopamine transporter
EPM: Elevated plus-maze
GDNF: Glial cell line-derived neurotrophic factor
GFAP: Glial fibrillary acidic protein
iNOS: Inducible nitric oxide synthase expression
Iba1: Ionized calcium-binding adapter molecule 1
NeuN: Neuronal nuclear antigen
NT3: Neurotrophin-3
IκBα: Nuclear factor of kappa light polypeptide gene enhancer in B-cells inhibitor α
NF-κB: Nuclear factor -κB
OF: Open field test
PD: Parkinson's disease
PBS: Phosphate-buffered saline
SEM: Standard error of the mean
SN: Substantia nigra
TBI: Traumatic brain injury
TH: Tyrosine hydroxylase
α-syn: α-synuclein.
